# A Radiological Clip Design Using Ultrasound Identification to Improve Localization

**DOI:** 10.1109/TBME.2024.3388203

**Published:** 2024-08-21

**Authors:** Jenna Cario, Zhengchang Kou, Rita J. Miller, April Dickenson, Christine U. Lee, Michael L. Oelze

**Affiliations:** Beckman Institute for Advanced Science and Technology and the Department of Electrical and Computer Engineering, University of Illinois at Urbana-Champaign, Urbana, IL 61801 USA; Beckman Institute for Advanced Science and Technology, University of Illinois at Urbana-Champaign, USA.; Beckman Institute for Advanced Science and Technology and the Department of Electrical and Computer Engineering, University of Illinois at Urbana-Champaign, USA.; Breast Imaging Department, Carle Foundation Hospital, USA.; Radiology Department, Mayo Clinic, USA.; Beckman Institute for Advanced Science and Technology, the Department of Electrical and Computer Engineering, and the Carle Illinois College of Medicine, University of Illinois at Urbana-Champaign, USA.

**Keywords:** Implantable medical devices, neoadjuvant chemotherapy, radiological clips, ultrasound identification

## Abstract

**Objective:**

We demonstrate the use of ultrasound to receive an acoustic signal transmitted from a radiological clip designed from a custom circuit. This signal encodes an identification number and is localized and identified wirelessly by the ultrasound imaging system.

**Methods:**

We designed and constructed the test platform with a Teensy 4.0 microcontroller core to detect ultrasonic imaging pulses received by a transducer embedded in a phantom, which acted as the radiological clip. Ultrasound identification (USID) signals were generated and transmitted as a result. The phantom and clip were imaged using an ultrasonic array (Philips L7–4) connected to a Verasonics^™^ Vantage 128 system operating in pulse inversion (PI) mode. Cross-correlations were performed to localize and identify the code sequences in the PI images.

**Results:**

USID signals were detected and visualized on B-mode images of the phantoms with up to sub-millimeter localization accuracy. The average detection rate across 30,400 frames of ultrasound data was 98.1%.

**Conclusion:**

The USID clip produced identifiable, distinguishable, and localizable signals when imaged.

**Significance:**

Radiological clips are used to mark breast cancer being treated by neoadjuvant chemotherapy (NAC) via implant in or near treated lesions. As NAC progresses, available marking clips can lose visibility in ultrasound, the imaging modality of choice for monitoring NAC-treated lesions. By transmitting an active signal, more accurate and reliable ultrasound localization of these clips could be achieved and multiple clips with different ID values could be imaged in the same field of view.

## I. Introduction

Breast cancer is among the most common cancers diagnosed in women in the United States, with an estimated 350,000 new cases in 2023 [1]. Patients with breast cancer metastasizing to the axillary lymph nodes generally undergo neoadjuvant chemotherapy (NAC) before surgery. NAC may induce a complete pathological response in 40–75% of node-positive cases [2]. Current surgical management after NAC involves targeted axillary dissection, where the sampled positive node is also resected during sentinel lymph node biopsy [3], [4]. As such, markers or clips implanted in the node during needle sampling later confirm that the positive lymph node was resected. In the setting of complete pathological response, however, the marked positive node is difficult to identify by ultrasound, the imaging modality of choice in the axilla, for preoperative localization [5].

Essentially, during treatment of node-positive breast cancer, two devices are percutaneously implanted: a marker or clip placed in the node at the time of needle sampling *before* starting NAC, and a localizer placed in the same node *after* NAC but before surgery. The majority of the over three dozen breast biopsy clips that are available for use during NAC are metallic, with recent designs employing nitinol to allow for expansion of the marker up to 10 mm once it is deployed from the needle. Despite newer and larger designs, these markers cannot be identified up to 50% of the time [5], [6]. This limits the success of ultrasound-guided preoperative radioactive seed localization of the marked node, as the marked target needs to be visible before deploying a low-dose Iodine-125 seed into the node. Various preoperative techniques to localize the marked nodes are available, including wire localizations, but they can only be performed on the day of surgery [7].

Several commercially available proprietary localization systems also exist and may be used as an alternative to preoperative strategies requiring same-day placement. However, these systems require clinics to purchase both the localizers and the imaging probes, which are not designed for compatibility with ultrasound systems. For example, the LOCalizer^™^ (Hologic, Inc.) is 11 mm long, 2 mm in diameter, and uses radiofrequency (RF) tags and a proprietary probe to identify these RF-enabled tags up to a depth of 6 cm [8]. Radioactive seed localizations, as mentioned above, additionally require a gamma-probe or Geiger counter for detection and must meet the requirements of the Nuclear Regulatory Commission. Another proprietary localization system is the Saviscout surgical guidance system (Merit Medical, South Jordan UT, previously Cianna Medical, Aliso Viejo, CA) which uses radar technology to achieve localization in a surgical setting. This localizer is 1.2 cm long and has two antennae. Lastly, the Magseed (Endomag, Cambridge, U.K.) requires constraints on surgical equipment when it is near the probe. The costs of these localizing methodologies involve the need for a particular detector, and in some cases, limit the diagnostic quality of other imaging modalities such as MRI, which is often a part of the standard of care in breast imaging in patients with breast cancer.

Improving visibility of objects in ultrasound images, particularly via designing powered devices, is an active area of research. Though not all designs are clips that remain in the body independently, commonalities arise in approach. For example, a configuration used by Guo, et al. for the purpose of improving visibility of surgical tools, such as catheters, bears a strong resemblance to the experimental setup that we independently developed and present in this work for radiological clips. In their work, pulses are generated from a small element on a tool’s tip and large pulses (≥20 V) were able to be returned to the imaging transducer from the transmitting element in order to form patterns and images [9]. However, this is not a device that would remain in the tissue outside of a surgical setting, so powering such a device is not of immediate concern. In the approach we introduce and for implantable clips broadly, power considerations remain at the forefront, which changes the trajectory of the work and experimental design. We focus on comparatively weak signals (voltages < 3.3 V) that are expected to be constrained by implantable device size in this concept’s ultimate form.

Ultrasound power harvesting is an attractive approach to powering because it can enable low form factor and long-term biocompatibility in fully-implantable devices which require little power. Multiple groups have investigated the use of incredibly compact ultrasound “motes” that harvest power from received ultrasound pulses and use the energy to manipulate backscatter, which can then be used to form a transmission channel between the probe and the mote [10], [11]. These approaches offer high localization accuracy and sufficient speed for real-time imaging. However, backscatter manipulation is fairly weak, and generally the effects are not highly visible in a traditional B-mode image. The motes themselves are also, due to their size, low in inherent visibility in a B-mode image, particularly *in vivo*. In the clinical setting free-hand scanning and patient movement can make B-mode detection for establishing power and device connections more challenging than in *in vivo* experiments with an anesthetized mouse and transducer fixed to a chemical stand [10].

Color Doppler twinkling has also been used as an ultrasound-compatible and non-powered device approach to identify some markers in preparation for insertion of a localizer. First described in 1996 by Rahmouni et al. [12], twinkling is described as rapidly fluctuating colors of an entity, such as kidney stones, during color Doppler ultrasound. The cause of twinkling is still under active investigation, but has been described in the work by Tan, Bi, and Ong [13] with the ULTRACOR TWIRL^™^ (Bard, Inc.) marking clip as well as other markers [14], [15]. The twinkle artifact appears a few millimeters below the clip and its effect can be enhanced by adjusting system settings. However, to the best of our knowledge, this strategy provides no real-time audio feedback on location and does not enable distinction between multiple tagged areas.

## II. Our Approach

In this work, we propose that the identification and localization tasks can be achieved by a single, ultrasound-compatible design, eliminating the need for localizers used specifically during resection and improving reliable visualization during and after NAC. In the proposed scheme, the radiological clip is a powered device, which transmits an ultrasonic signal from a piezoelectric element when imaged by an ultrasound probe. The signal encodes information to achieve ultrasound identification, i.e., USID, and aids in locating the clip itself by adding an additional visual effect in the beamformed data. The imaging system is programmed with additional software or subroutines to detect this visual effect and identify the encoded identification information within the received signal. This information can be further used to provide audio feedback on the probe’s proximity to the clip to be used in surgery.

This clip design requires four major components: a piezoelectric element that can receive and transmit electrical signals through tissue as acoustic pressure waves, a means of triggering a circuit that generates a USID signal, a means of ensuring that the transmitted USID signal does not re-trigger the circuit, and a means of powering the circuit. The overall circuit must be small in size. The small profile allows clips to mark comparatively smaller lesions and be inserted in a minimally-invasive manner. The power source must fit the desired size profile and enable intermittent imaging over several months of NAC, and allow the clip to be imaged and identified afterward during resection. This power source may be either a battery or energy harvested from the imaging probe. Components with low power draw and effective power management are necessary to achieve this requirement. The clip will also need to be able to be encased in a biocompatible shell, conformal coating, or outer layer without major reductions in signal quality or directivity that would inhibit localization. In this work, we evaluate the components needed to achieve the desired functionality in a scalable manner to meet the subsequent size, power, and biocompatibility requirements. However, in this study we do not build an electronic clip at a scale that the clip can be inserted into humans.

The USID signal itself is also subject to design constraints, and an appropriate USID signal is one that satisfies three requirements. First, it must have an encoding scheme with sufficiently fast decoding so visual and any audio feedback can remain clear and approximately real-time. Second, the signal must be tunable, e.g., multiple USID signals can be generated from a singular design with minimal alteration. Finally, the signal must be long enough to successfully encode information and be detectable, but short enough to be matched with the acquisition rate of an ultrasound imaging system.

The design we present uses a 64-bit pseudo-noise (PN) code as the USID signal. This scheme is chosen because it can be fetched from a stored code library in read-only memory (ROM) with minimal processing prior to transmit. A 64-bit code transmitted at 4 MHz in tissue has an approximate length of 16 *μ*s. For comparison, the acquisition window used in our experiments is 112 *μ*s. Importantly, PN codes tend, with increasing sequence length, toward having an impulse response at zero lag when auto-correlated. This allows for clear and distinct localization, as a small codebook can be kept by the ultrasound system and decoded quickly via fast Fourier transform (FFT) to cross-correlate the possible codes and the received data. Because PN codes are pseudorandom sequences, there is a possibility that parts of selected codes may cross-correlate strongly with other codes at shifted temporal locations. As such, use of PN codes also necessitates verifying that the autocorrelations of the selected codes are able to be distinguished in magnitude from the cross-correlation of each code in the library with each other code in the library at any temporal shift.

Both the background tissue signal and the systems used to perform imaging can vary greatly. To further increase the signal-to-noise ratio (SNR) of the clips and mitigate the effect of these variables, pulse inversion (PI) imaging is used. Conceptually, pulse inversion imaging arises in ultrasound from modeling the relationship between pressure and density of an acoustic wave in the imaging medium as a Taylor series and considering only the first- and second-order responses, which represent the fundamental and second harmonic, respectively. When the echoes generated by two pulses of identical shape and opposite polarity are summed together, the linear, first-order response vanishes; conversely, the nonlinear, second-order response will be doubled in magnitude. Other, higher order terms that are excluded will follow a similar pattern of even harmonics doubling and odd harmonics vanishing. Typically, PI imaging is conducted at transmit strengths that allow energy to be transferred into higher harmonics to improve image quality over an acquisition using solely the fundamental frequency. However, at lower transmit strengths, tissue response remains approximately linear. As a result, the tissue signal can be greatly attenuated by PI imaging.

The USID circuit only uses the incoming imaging pulses as a trigger to begin signal transmission, so the pulse polarity has no effect on the circuit’s response provided both the positive and negative polarity pulses can trigger the circuit at approximately similar locations along their temporal axes. Consequently, the USID signal will appear nonpolar in PI imaging conditions and will resemble a second-order response. When the two acquisitions are summed, the USID signal strength is effectively doubled while the linear tissue signal is removed. PI imaging can therefore be used to isolate the USID signal from the surrounding tissue and the linear tissue signal.

Additionally, if one acquisition is subtracted from the other instead of summed, the nonlinear terms will vanish and the linear tissue signal will be amplified. If these subtracted data are beamformed and presented onscreen, portions that might otherwise be obscured by the USID signal will be visible, and the summed data necessary to identify and localize the USID clip can be processed in the background.

In this paper, we have focused on the basic design elements necessary to test these individual facets in tandem. In particular, we devised a custom printed circuit board (PCB) platform mimicking the intended hardware and transmit/receive functionality of a USID clip, equipped with USID codes designed to increase clip visibility and provide identification. We also created the code necessary for an ultrasound imaging system to be able to decode this information upon receipt. A preliminary version of this work has been reported [16].

## III. Methods

The experimental setup consisted of two parts: 1) the Verasonics research imaging system (Verasonics, Kirkland, WA, USA), its code, and the attached imaging probe, and 2) the circuitry used to simulate the USID clip. These two halves interfaced in an imaging medium via ultrasound. The setup was tested using three individual tissue-mimicking phantoms made of agar and having 70 to 90-*μ*m glass bead scatterers distributed uniformly throughout with spatially random locations. A 1-mm, epoxy-coated microcrystal (Sonometrics Corp., London, Ontario, Canada) serving as the clip’s piezoelectric element was embedded into the center of these phantoms at two depths. The first and shallower of the two depths was approximately 10 mm from the surface of the phantom, and the second, deeper location was approximately 25 mm from the surface of the phantom. Actual imaging depth varied between 10- and 30-mm between phantoms and imaging trials, the latter due to different amounts of coupling gel between trials. The choice of these depths was based on a previous study that examined 524 clip placements in the breast and found that the range of depths of the clips from the skin surface as observed using ultrasound was between 0.4 to 2.5 cm with an average depth of 1 cm [17].) In order to visually compare the USID clip signals with a passive commercial clip, a Tumark^™^ Eye clip (Hologic Inc., Marlborough, Massachusetts, USA) was included in the phantoms. Its depth in the phantom ranged between approximately 15–25 mm from the phantom surface.

### A. USID Clip Circuit

**Fig. 1.**
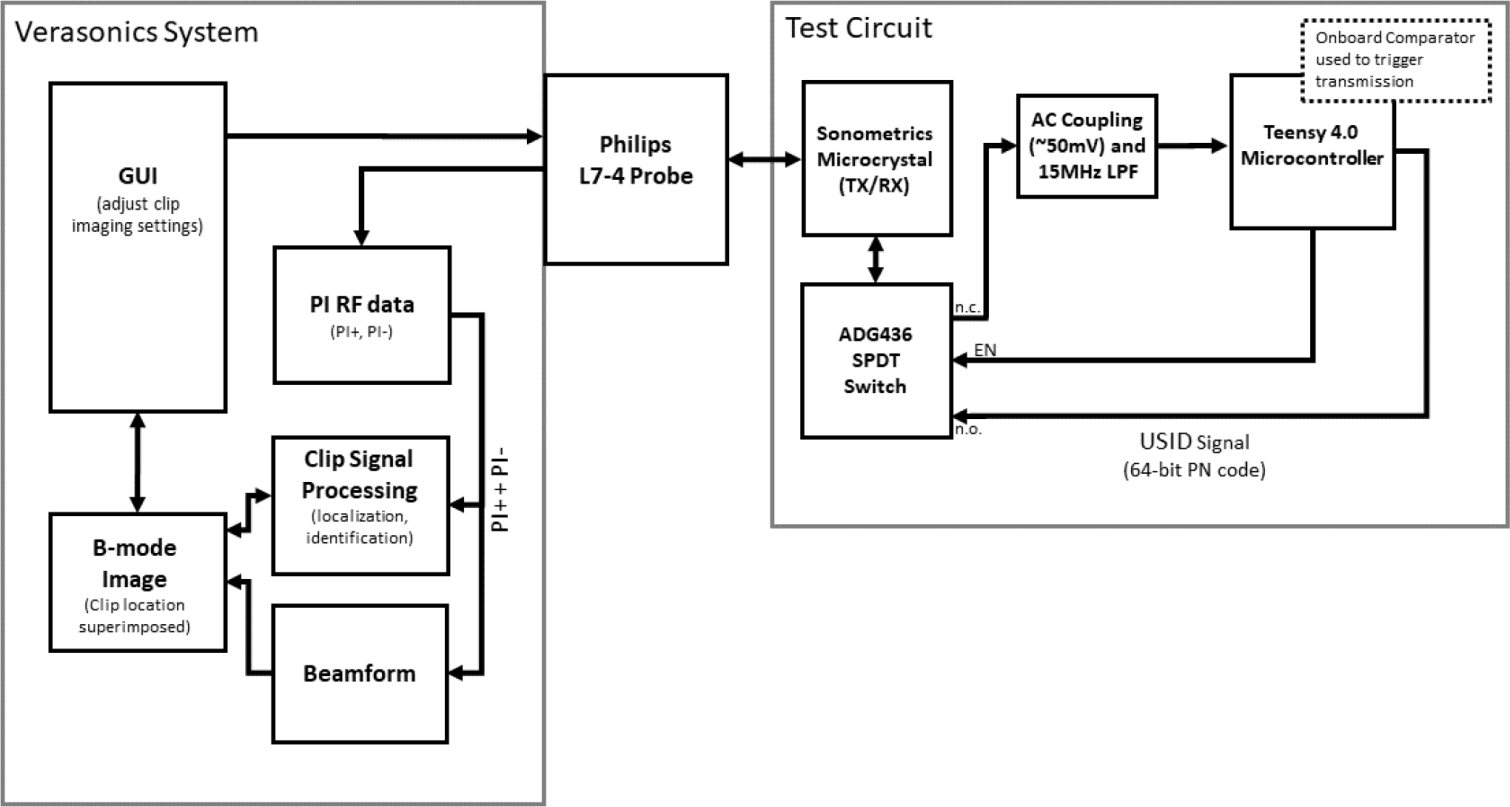
Diagram describing the flow of signals and data through the USID system.

**Fig. 2.**
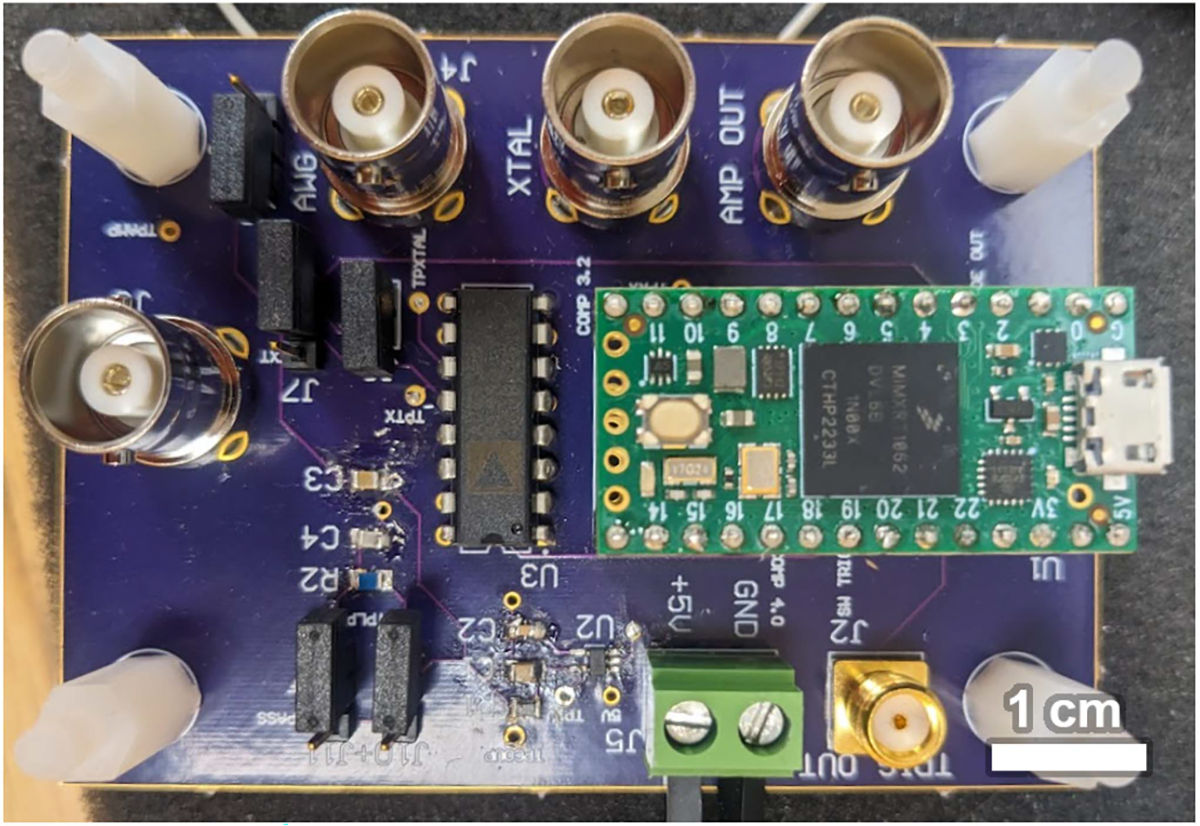
Photograph of PCB used to test the USID hardware concept. One PCB was used to control one crystal.

Several iterations of USID clip circuitry were tested as the system developed, with the system flow for the experimental configuration described herein illustrated in Fig. 1. At this stage of development, the PCB served as the test bed platform used to evaluate the USID clip concept and generate test USID signals. To this end, this PCB, measuring 79 mm by 58 mm, was used to interface a Teensy 4.0 microcontroller (PJRC, Sherwood, OR, USA) with the microcrystal via BNC cable in order to receive, process, and transmit signals. An ADG436BNZ (Analog Devices, Inc., Wilmington, MA, USA) single-pole, double-throw (SPDT) analog switch was used to route the input and output through a single crystal and to the appropriate Teensy pins. The Teensy 4.0’s core processor and its four onboard analog comparators were clocked at 600 MHz. A photograph of this circuit board is shown in Fig. 2.

The received signal, which could contain an ultrasound imaging pulse, was used as a digital interrupt for the Teensy code. The received signal was filtered through a single-stage RC low-pass filter having a cutoff of 15 MHz to attenuate high-frequency noise. This filtered receive signal generated a software interrupt via one of the comparators, whose expected sensitivity was 52 mV as determined by 64 taps (6 control bits) of the 3.3 V reference voltage. When the interrupt subroutine attached to the comparator was triggered by a voltage change resulting from a received ultrasound pulse, the Teensy transmitted the selected USID code, stored in an array in memory, via a for-loop. This bit-banging solution used \nop commands, which execute no operation for a single execution cycle, to create a pulse train with a repetition frequency of 3.90 MHz (approximately matching the center frequency of the imaging probe) and a duty cycle of 33%. Once generated, the received signal was passed through the SPDT switch to the microcrystal via a wire lead connected to the PCB and into the imaging medium. The transmitted output ranged between 0 and approximately 2.5 V.

### B. Verasonics System

The Verasonics research imaging system leverages MATLAB (Natick, MA) to enable user modification of the data-acquisition process. Example code included with the system for PI imaging was used as a template for code to image the USID clip. The code was modified to image with a Philips L7–4 probe (Philips Healthcare, Bothell, WA, USA), a 128-element linear array chosen for the bandwidth overlap shared with the sonomicrocrystal used in the USID circuit. The L7–4 probe fired all elements simultaneously in a single plane wave for each acquisition. Every other acquisition used a pulse that was of opposite polarity to the previous excitation pulse to achieve PI imaging. The pulse repetition frequency (PRF) between two pulses was automatically determined by the imaging system based on the maximum imaging depth. PRF and frame rate were ultimately determined by the total processing time for beamforming and the USID-specific functions added to the code.

A custom delay-and-sum subroutine leveraging GPU capabilities was used for beamforming. A second custom subroutine was created to localize and identify the USID signals (IDs) that might appear in the beamformed data. The chosen localization approach used normalized cross-correlations, which were computed for each of the 128 lines of data, with the potential codes in the codebook. We recorded the actual transmitted codes from the circuit on an oscilloscope, and these recorded waveforms were used as the reference waveforms in the cross correlation. Oscilloscope data of the transmitted codes improves cross-correlation over what would be possible with the “ideal” code by capturing the impulse response of the circuit system. An “ON threshold” for the cross-correlation magnitude served as the lower bound for the presence of a USID signal.

In one operational mode, a specific USID clip can be selected and the imaging system would then look for that code. The USID value of interest was selected on the Verasonics system. The subroutine would determine laterally where this ID’s cross-correlation reached its maximum, and if the cross-correlation magnitude was greater than the ON threshold. If so, a detection occurred. On the line containing this maximum response, the axial depth having maximum cross-correlation was used to obtain the location of the USID signal’s origin. In this manner, both axial and lateral positions of the USID clip were identified in an image frame.

Alternately, the operator could select to detect any clip signal and identify and localize it. In this mode, dubbed “freewheeling” mode, the beamformed data were cross-correlated with all possible codes. The code having the highest cross-correlation that crossed the ON threshold was selected as the USID, with otherwise identical steps for lateral and axial localization. In this mode, multiple USID clips could be identified and displayed if they were in the field of view.

### C. Testing the Clips

The Verasonics system’s user interface has several parameters of interest that were fixed for the tests of the clips and code. These included the time-gain compensation (TGC) left at the default settings and imaging pulse voltage at 13.7 V. This voltage was observed as the lowest step able to trigger the crystal reliably. The ON threshold was set to 0.3 normalized units, where normalization is relative to the geometric mean of the cross-correlated signals’ autocorrelations at zero lag. This value was selected based on observations of the system operating in conditions with IDs absent, a single ID present in both selected-ID and freewheeling modes, and two different IDs, with one selected for localization. Test variables included the USID to search for and the depth of the signal. These were used to assess localization accuracy.

Acquired RF data and beamformed data were saved with relevant system parameters that could be used in post-processing to reconstruct the localization and identification process as needed. In each acquisition, 100 frames of data were gathered. USID data (x- and y- coordinates and ID value) as calculated by the system were also saved. Frames where an ID could not be detected were indicated in this saved data. Localization accuracy over 100 frames was tested for each ID at both possible depths, resulting in 1600 frames of data (8 IDs · 2 depths · 100 frames = 1600 frames). These acquisitions were repeated over *n* = 19 independent system trials (30,400 total frames, 15,200 per depth). Data from a second, faulty crystal were excluded. Additionally, each frame’s processing was independent of every other frame’s.

Out-of-plane behavior was also studied by acquiring scans of the deeper crystal as the probe was translated across the surface of the phantom with a Daedal micropositioning system (Daedal, Inc., Harrison City, PA, USA). Approximately 100 frames of data for each ID were gathered at 40 *μ*m increments, resulting in a total translation window of approximately 4 mm.

**Fig. 3.**
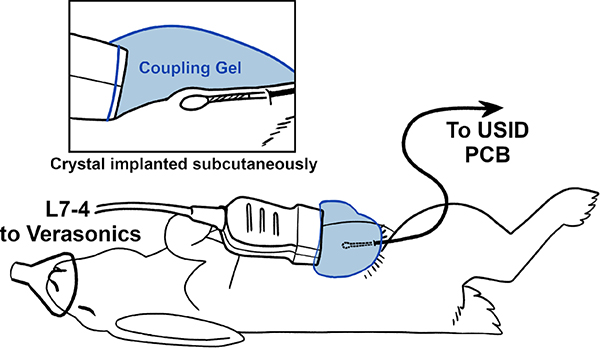
Diagram showing scan setup for acquisition of *in vivo* data.

One *in vivo* trial with a female 5-month-old New Zealand White rabbit (Charles River Laboratories, Wilmington, MA) was performed according to a protocol approved by the University of Illinois at Urbana-Champaign Institutional Animal Care and Use Committee (IACUC protocol 21190). The rabbit was anesthetized with 2% isoflurane, the skin over the mammary fat pad was shaved and disinfected, and the microcrystal was implanted subcutaneously. The crystal was connected to the USID PCB and imaged using the Verasonics system for transmission of IDs 1–4. A total of 100 frames of data were recorded for each ID tested. A diagram of the experimental setup is shown in Fig. 3. In order to visualize the crystal, the probe was held at a very shallow angle relative to the skin (less than 45°).

(1) $$SNR=10\;{log}_{10}\left[\frac{\sigma_{s+n}^{2}}{\sigma_{n}^{2}}-1\right]$$

The SNR for each ID at each depth was calculated according to (1) [18][19], where $\sigma_{s+n}^{2}$ is the variance of the data containing the USID signal plus background noise and $\sigma_{n}^{2}$ is the variance of the background noise without the USID signal. For each ID at both depths, 100 frames of beamformed data were averaged into one image. Variance was calculated laterally along the line of the ground truth signal origin and over the axial range visually determined to contain the USID signal. The background signal was calculated as the variance of an axial line with the same length as that sampled for the signal data but laterally shifted 20 lines toward the center of the image to avoid capturing the fan-shaped tail of the USID signal.

The capabilities of the “freewheeling mode” were also tested in post-processing, though the code on the Verasonics system for real-time visualization was identical. All 1,900 frames of data for each ID at both depths were processed using the same ON threshold as was originally used for data collection. The IDs calculated for each detected signal were recorded.

## IV. Results

Under PI imaging conditions, the USID signal was visible as a fan-shaped tail behind the crystal. Signal transmission was verified via oscilloscope to be dependent on the receipt of an imaging pulse through the crystal and on no other factors present in the experimental setup. In some frames, the second of the two pulses would not trigger or would trigger with a longer delay than the first due to jitter in the analog comparator. However, the strength of the transmitted signal tended to be sufficient that these errors did not have an adverse effect on signal visualization or localization.

To estimate the pressure levels transmitted by the ultrasound array and received by the clip crystal, a 75-*μ*m needle hydrophone (Precision Acoustics, Dorcheseter, U.K.) was used to measure the pressure levels near the elevational focus of the array, i.e., 25 mm. A peak pressure of 140 kPa was measured in water for an excitation voltage of 13.7 V. Therefore, an approximate maximum of 1.04 mW of power was delivered to the crystal from the imaging probe. The next lower voltage step (11.3 V) corresponded to a peak pressure of approximately 110 kPa in water and was less reliable in triggering the transmission of USID signals from the crystal in the phantom.

**Fig. 4.**
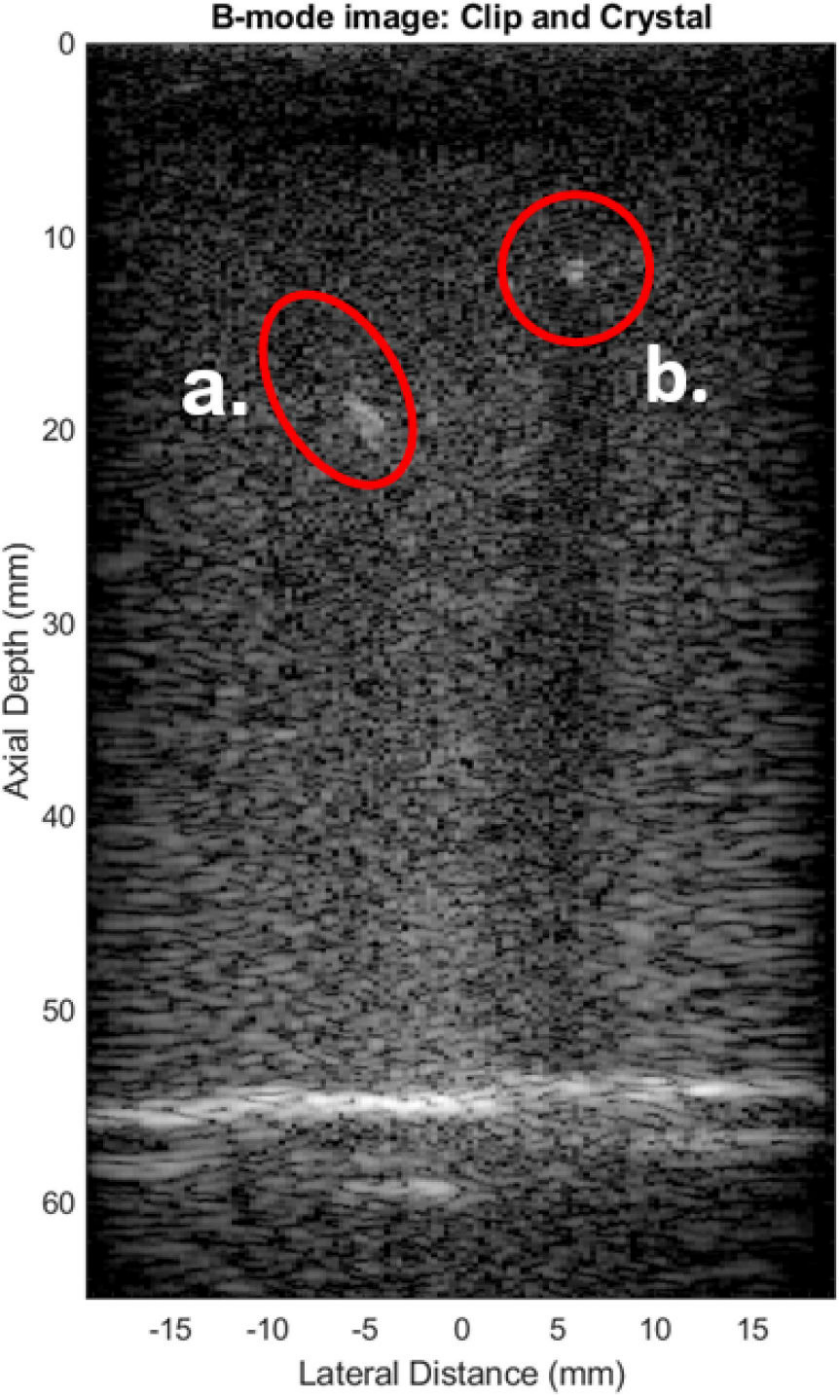
B-mode image, with the Tumark Eye clip (a.) and 12-mm-deep clip (b.) circled.

Fig. 4 shows an annotated view of a B-mode image of one tissue-mimicking phantom, containing the shallow crystal and the metal clip. This view was displayed without using PI imaging and with no USID signals being transmitted. The bottom of the phantom at approximately 55 mm deep appears brighter than either the clip or the crystal.

The SNR (see Table I) calculated for the IDs varied with signal transmission depth, with the shallow clip providing a signal strength averaging about 2.75 dB less than that achieved for the deeper clip placement. While SNR was found to be significantly different between the two depths at a 95% confidence interval (CI) ([11.29, 12.25] dB for shallower crystal and [14.30, 14.75] dB for deeper crystal), it should be noted that time-gain compensation (TGC) was enabled for all scans, with increasingly lower sections of depth having higher gain.

**Fig. 5.**
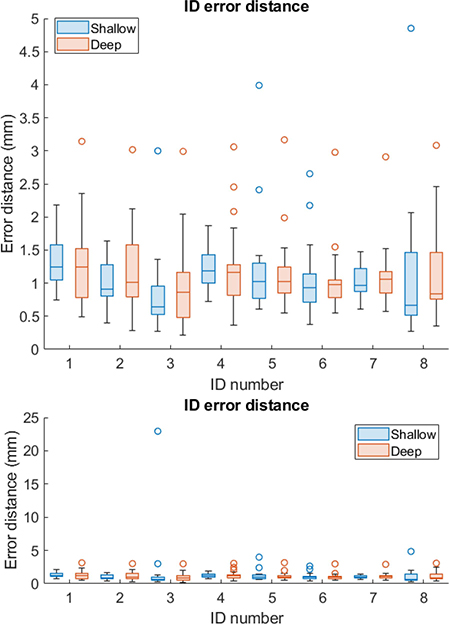
Box plots showing localization distance results for *n* = 19 trials of each ID at both depths. The lower plot shows an outlier trial for ID 3, shallow depth.

Error distance across all trials is shown in Fig. 5 in box plot form. The trials are grouped by depth (in color) and ID along the x-axis, while the mean error distance across each of the 19 trials is shown on the y-axis. Excluding shallow depth trial 4 for ID 3, the mean error distance for all trials is less than 5 mm, and the median value across 19 trials less than 1.5 mm for all IDs and depths. The deep trials appear to have more outliers, marked as empty circles, than the shallow trials.

**Fig. 6.**
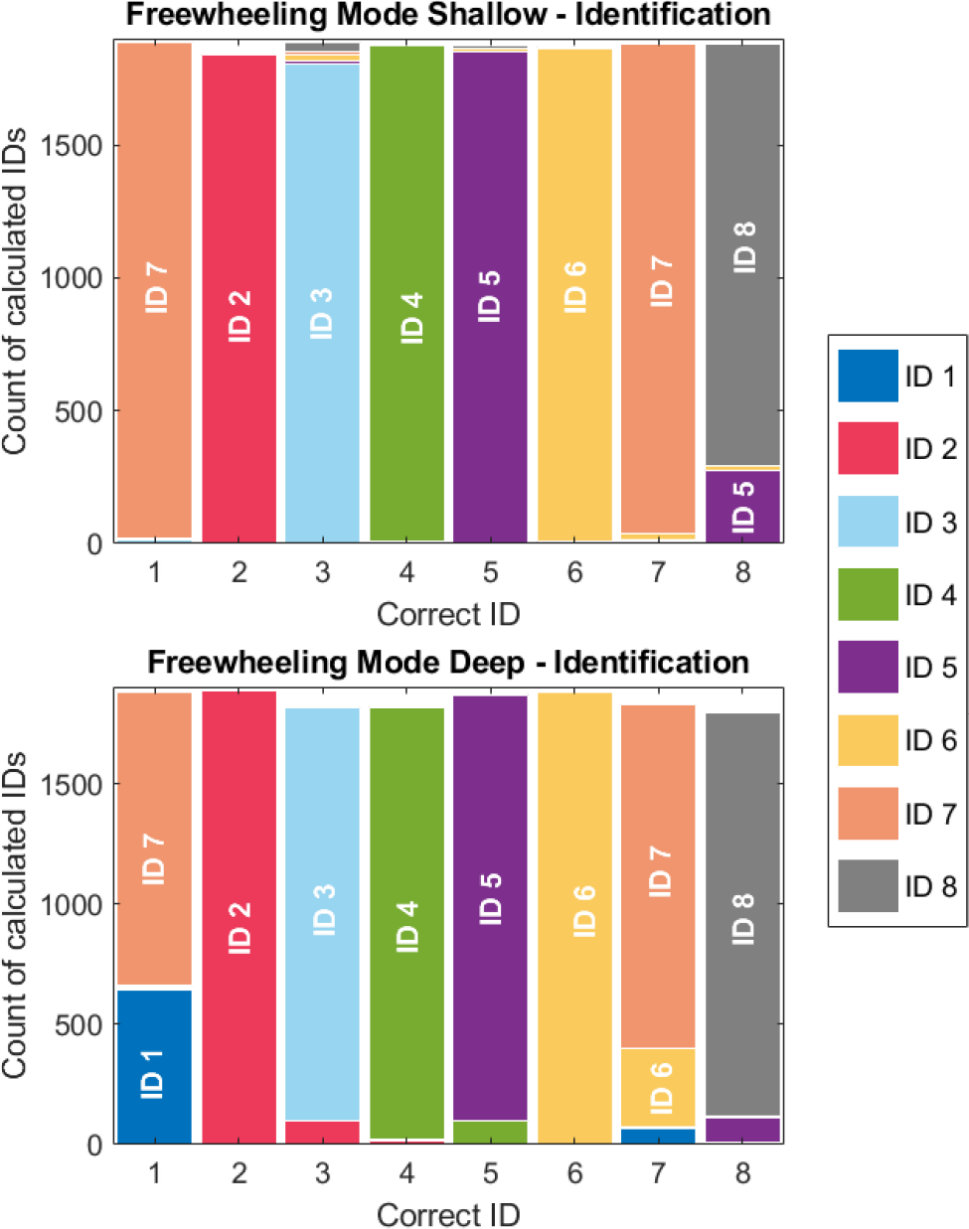
Bar graphs of calculated ID against the transmitted ID value.

For “freewheeling mode”, identification needs to be tested in addition to localization. Fig. 6 shows how different IDs were identified. The bars of this chart are the true value of the transmitted ID, while the counts indicate the calculated ID value, summing up to the number of successful detections per ID out of a total of 1900 frames each. Detection rates and identification accuracy remain generally similar at both depths. The overall detection rate across all trials, IDs, and depths was 0.9811 (95% CI [0.9740, 0.9882]). Notably, ID 1 was confused for ID 7 at both depths and was almost always identified as such in the shallow-depth trials. A non-negligible cross-correlation exists between ID 1 and ID 7. ID 8 was occasionally misidentified as ID 5 at both depths. IDs 2, 4, and 6 were identified correctly in nearly all transmissions, regardless of depth.

As the imaging plane was translated across the crystal at 23-mm deep, the USID signal would begin transmission, persist for approximately 60 frames, and then cease transmission. This corresponds to a translation distance of approximately 2.4 mm (the L7–4 probe has an elevational aperture of 7.5 mm). The epoxy coating encapsulating the 1-mm piezoelectric crystal element was measured as having a diameter of 2.43 mm. When the crystal was mostly in-plane, for approximately 10 frames (0.4 mm), the USID signal appeared brightest and localization was observed to be most accurate. Less optimal alignment of the imaging plane and crystal, which still produced a USID signal, was not as accurate at detection or localization. Across each ID, the locations of turn-on and turn-off of the USID signal were within 5 frames of each other, though the bright USID signal and better localization persisted intermittently beyond 10 frames for some of the IDs.

In the supplementary materials provided with this text, we provide animations showing the performance of the USID localization and identification system in standard mode, querying ID 8 while IDs 8 and 2 transmit from 12- and 27-mm deep, respectively, using a second crystal embedded simultaneously. Subtraction of the two PI acquisitions is used to display the B-mode image animation. A rudimentary version of localization of multiple IDs simultaneously, using the same data, is also provided. Erroneous localization in standard mode is limited within these 100 frames of data, and though multiple IDs may be erroneously identified in a small region due to errors in code, both IDs were captured. No crosstalk was observed between the two crystals.

**Fig. 7.**
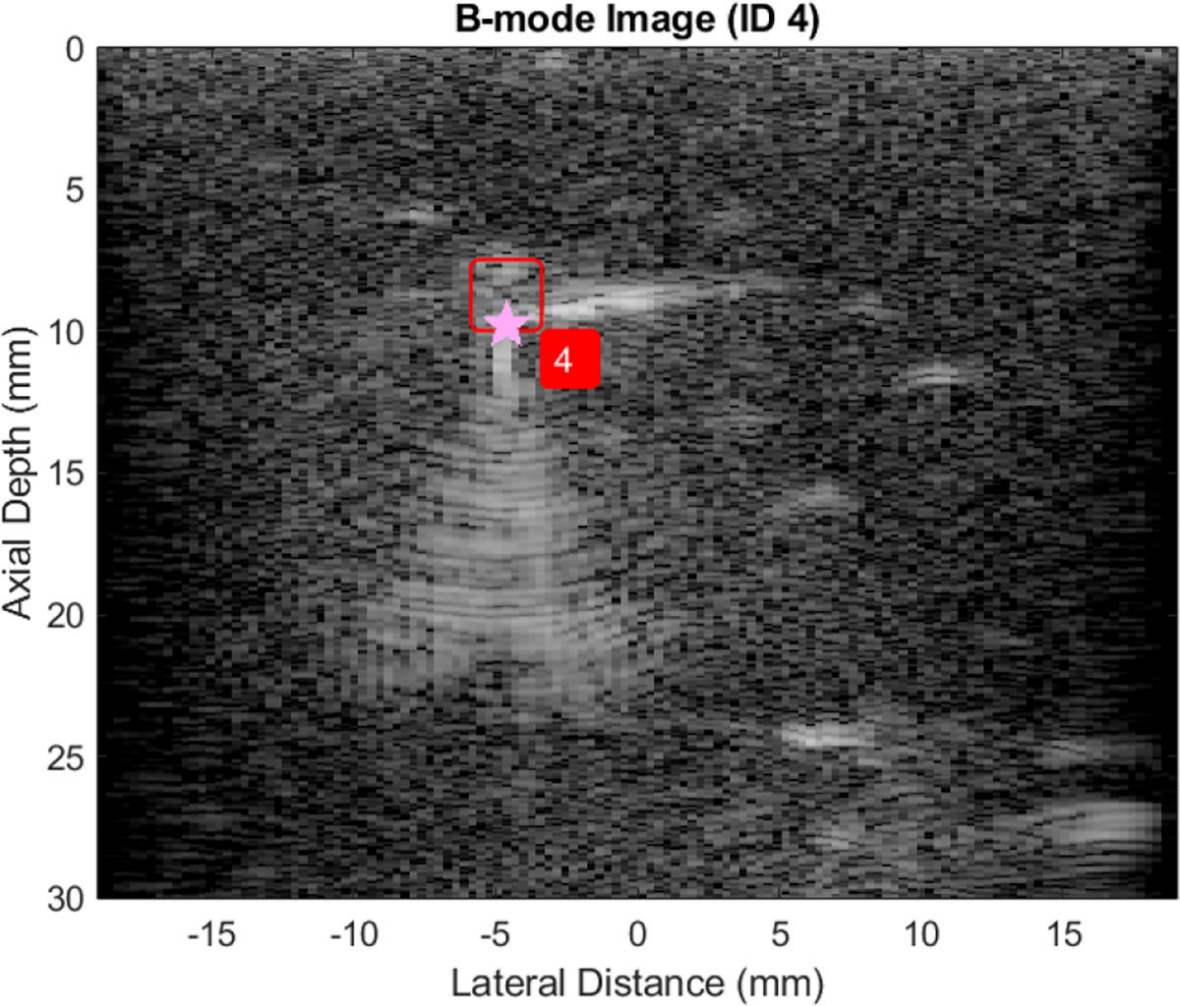
Example frame of *in vivo* data (cropped to a maximum depth of 30 mm) showing USID signal transmission. The ground truth location of the clip is marked with a pink star.

In the *in vivo* data presented in Fig. 7, we show successful transmission, localization, and post-processing identification of the USID signal. The number of successful triggering events across the four trials was 50 (of 400 frames) and 249 frames had no apparent triggering, which could occur if the image plane did not intersect the clip. In the remaining frames, triggering occurred at least once per frame, but was offset axially by more than a millimeter. The clip was located approximately 10 mm from the probe and 2 mm from surface of the skin, though no other major anatomical features were visible due to the shallow imaging angle.

## V. Discussion

Fair accuracy was observed in localization of the clip signal for nearly all phantom trials. Mean error in localization distance was less than 5 mm for nearly every ID-depth pair across all trials, and an overall mean error distance of 1.186 mm over 304 trials was observed, which suggests that the clip could be localized successfully. This metric of success is based on clinical implications of having errors in localization. To be successful, localization should be able to overlap the clip along its smallest dimension. Given that the clips are intended to be small and inserted with a needle, the goal is to create a clip design with a smallest dimension no larger than 2 mm.

In this system, error distance variance is an indicator localization stability. If variance is high, this implies that the localization is jumping between points that are widely spaced in the image. An “acceptable” variance would be arbitrary to set without considering the associated error distance, but it can indicate data sets in which such jumping behavior is problematic. For example, trial 4 of ID 3 at shallow depth was a notable outlier in error distance. It was observed that in this trial, localization jumped between approximately the true location of the crystal at 12 mm and a point 50-mm deep. This effect depended on the strength of the USID signal in the frame, which flickered in intensity. The variance associated with the error distance in this trial was 348 mm^2^, though the variances for most other trials tended to be below 1 mm^2^ or 0.1 mm^2^. Other outlier trials (as marked in the box plot) had associated error distance variances of 150 mm^2^ (ID 8, shallow), 0.0132 mm^2^ (ID 4, deep), and 112 mm^2^ (ID 5, shallow).

Detection rates were found to be acceptable for the localization process, with a final overall detection rate across every ID, depth, and trial calculated at 98.1%. The two depths, when compared, did not have a statistically significant difference in detection rates at a 95% confidence level (CI [−0.009792, 0.02359]). False positive rates were higher than expected across 24 independent acquisitions of the crystals with no USID signal present (95% CI [0.05017, 0.3506]) but still represented a statistically significant difference from true positive detection at the given threshold (95% CI [0.9740, 0.9882], *n* = 304). Changes to ON threshold may be leveraged to decrease false positive rates in future system iterations. The detection rates presented here may represent a best-case scenario, as the codebook was collected from received data and hypothetically perfectly matched to the impulse response of the transmitting crystal. In future iterations, this will be corrected by forming the codebook from the responses of multiple crystals.

In the *in vivo* validation of the USID concept, subcutaneous implantation of the crystal and its wire lead meant that crystal orientation was always parallel to the skin, increasing the difficulty of imaging and triggering the USID signal transmission despite only thin tissue between the probe and the crystal. Incorrect triggering events occurred frequently, possibly due to tissue motion, signal interference, or strong reverberant echoes. In future *in vivo* experiments, we will image within tumor models that will enable better alignment between the probe and clip while providing additional tissue layers to image through.

The localization and identification procedures reduced the frame rate of the B-mode imaging due to heavy processing loads for cross-correlation. The original frame rate around 30 frames per second recorded without additional algorithms was reduced to between 6 to 12 frames per second. Frame rate started out on the higher end of this range, but reduced to approximately 6 fps over the course of active imaging, suggesting additional code refinement can achieve better frame rates that may be more acceptable in a higher-motion clinical environment.

The hardware used in this test platform was much larger than would be used in a clinical setting, and requires lead wires for power supply to the board, which is not feasible in practice. Power and biocompatibility considerations for the clip design are also important for clinical translation. Ultrasound power harvesting may be viable, particularly as the necessary USID functionality (including power management) could likely be accomplished within a 500-*μ*W power budget in a 16-nm-process application-specific integrated circuit (ASIC). Such a circuit would also have the advantage of a small footprint, i.e., area less than 1 mm^2^. We expect that the use of a conformal coating such as parylene-C, which is commonly used for other implantable electronics, may also be used for the clip to ensure biocompatibility.

One shortcoming of this work was the selection of the microcrystal, which had a center frequency of only 1.2 MHz. This off-the-shelf product functioned for this work but was not optimally matched to our needs. Furthermore, one crystal originally used in our experiments was found to be faulty after repeated wear and was replaced by the single crystal from which all data in this manuscript was gathered. We intend to have custom piezoelectric elements with matched impedance, lower directivity, and higher center frequencies for use with future iterations of the clip. We expect the center frequency of these elements will still be somewhat low-frequency, e.g., 4–5 MHz, relative to more typical frequencies for breast imaging, e.g., ≥12 MHz. This choice is justified to maintain higher penetration depth and for improved power harvesting.

Preliminary tests capturing the signal input/output, detection, and memory storage portions of the circuit in 65-nm process IC technology required approximately 250-*μ*m^2^ of chip area. The elements of ASIC design not addressed in the preliminary USID may take the remaining space on the 1-mm-square die. A smaller process such as 16-nm is intended to be used with a final prototype, so the footprint taken by the presently-modeled circuitry would be comparatively smaller and open more chip area for the design elements not yet addressed. Overall device footprint would be influenced by storage capacitor size, crystal size, USID IC size, and connections between these elements and external programming such as the memory address of the ID on a given clip. Capacitance for a storage capacitor can be varied within a standard package size, such as 0603, which has a footprint approximately 1.55 mm by 0.85 mm. The crystal would be no larger than 1 mm by 1 mm. The PCB itself would need to fully encompass each of these devices and would likely be approximately 1.57 mm thick and the thickest component in the design. We predict that these form factors will be sufficient to attain miniaturization for implantation.

## VI. Conclusion

USID signals, which encoded identification information using 64-bit PN codes, were transmitted acoustically from a custom PCB and decoded by the receiving ultrasound system. Cross-correlation was used to successfully localize and identify the signal origins in beamformed B-mode data within 2 mm of their actual position. Localization was accomplished in real time, with frame rates between approximately 6 and 12 fps. The detection rate for the eight tested IDs, tested at depths of approximately 10 mm and 25 mm, was 98.1%. We envision an implant prototype with a length of no more than 12 mm, and expect that our current level of localization accuracy and detection capability can, through addressing shortcomings we have identified, be improved to a level that is sufficient for both visual localization during NAC as well as for retrieval during surgical procedures.

## Supplementary Material

supp2-3388203

supp1-3388203

## Figures and Tables

**TABLE I:** SNR Value, in Db, for Each ID Transmitted From Both Depths

ID	SNR, Shallow (dB)	SNR, Deep (dB)

1	11.908	14.337
2	11.884	14.810
3	11.536	14.323
4	12.417	14.504
5	11.661	14.941
6	11.864	14.628
7	11.776	14.657
8	11.112	13.993
